# Sensogenomics and the Biological Background Underlying Musical Stimuli: Perspectives for a New Era of Musical Research

**DOI:** 10.3390/genes12091454

**Published:** 2021-09-21

**Authors:** Laura Navarro, Federico Martinón-Torres, Antonio Salas

**Affiliations:** 1 Unidade de Xenética, Instituto de Ciencias Forenses (INCIFOR), Facultade de Medicina, Universidade de Santiago de Compostela, Galicia, 15706 Santiago de Compostela, Spain; Laura.Navarro.Ramon@sergas.es; 2 GenPoB Research Group, Instituto de Investigación Sanitaria (IDIS), Hospital Clínico Universitario de San-tiago (SERGAS), Galicia, 15706 Santiago de Compostela, Spain; 3 Genetics, Vaccines and Pediatric Infectious Diseases Research Group (GENVIP), Instituto de Investigación Sanitaria de Santiago (IDIS), Universidad de Santiago de Compostela (USC), Galicia, 15706 Santiago de Compostela, Spain; federico.martinon.torres@usc.es; 4 Translational Pediatrics and Infectious Diseases, Department of Pediatrics, Hospital Clínico Universitario de Santiago de Compostela (SERGAS), Galicia, 15706 Santiago de Compostela, Spain

**Keywords:** genomics, transcriptomics, gene expression, sensogenomics, musical stimuli

## Abstract

What is the actual impact of music on the human being and the scope for scientific research in this realm? Compared to other areas, the study of the relationship between music and human biology has received limited attention. At the same time, evidence of music’s value in clinical science, neuroscience, and social science keeps increasing. This review article synthesizes the existing knowledge of genetics related to music. While the success of genomics has been demonstrated in medical research, with thousands of genes that cause inherited diseases or a predisposition to multifactorial disorders identified, much less attention has been paid to other human traits. We argue for the development of a new discipline, sensogenomics, aimed at investigating the impact of the sensorial input on gene expression and taking advantage of new, discovery-based ‘omic’ approaches that allow for the exploration of the whole transcriptome of individuals under controlled experiments and circumstances.

## 1. Introduction

What is the actual impact of music on the human being? Any analysis of the relationship between music and human biology, physiology, or psychology begins by highlighting the universal association between music and any civilization. According to Zatorre and McGill [1], music and, more generally, art transcend mere perception because they contact our more primordial neurobiology. However, what is the scope for scientific research in this field?

It is well-known that engaging in active music learning over a sustained period generates long-term, measurable physical, psychological, and sociocultural benefits for the individuals and groups involved. The scientific evidence of music’s value (from clinical science, neuroscience, and social science) keeps increasing [2]. However, compared to other areas of biology, the study of the relationship between music and human beings has been limited: only psychology and, more recently, neurosciences have investigated our biological relationship with music in depth.

One can intuitively understand why we are driven to eat, drink, have sex, talk, etc., based on the uncontroversial adaptive functions of these impulses; however, the drive to engage in music, a compulsion that is arguably just as pervasive in our species, has no such intuitive explanation [3]. Listening to music is amongst the most rewarding experiences involving the dopaminergic system, since it increases cerebral blood flow in specific regions [4]. Furthermore, genomic studies support the role of the dopaminergic pathway and its link to the reward mechanism as a molecular determinant in positive selection of music [5]. Music triggers reward systems, similar to food, drugs, or sex; for this reason, it can be very important for maintaining physical and mental health [6]. Overall, the seeming benefits of music on health explains the growing interest of using it in clinical settings [7,8].

At the same time, advances in the field of health and understanding of the biological bases of the physical (phenotypic) characteristics in humans are progressing at an extraordinary rate. To a large extent, this is due to the enormous development that genomics (and the “-omic” sciences, in general) and molecular technologies are undergoing, as well as the cost reduction of these techniques. As such, during the last two decades, progress has been made in understanding the genetic basis of a good number of common diseases (Parkinson’s, Alzheimer’s, schizophrenia, cancer, etc.) but also in our appreciation of the biological bases of human behavior or the ability of people to acquire knowledge. As a result of this research, a large number of genes, many involved in various neurobiological pathways, have been described.

Even so, genetics have contributed only very discretely to the understanding of the biological bases of music; the genetic architecture underlying music-related skills is largely unknown, remaining a field that is still in its infancy [9,10]. The arrival of new genotyping and sequencing genomic technologies provides us with great opportunities to further investigate this field. However, only a few studies have been devoted to the analysis of gene expression regarding musical stimuli.

Here, we first review scientific progress, in two main research strands: (1) the genetic basis of music in human evolution (Section 3) and (2) the impact of musical stimuli in our genome (Section 4). Thus, Section 3 focuses on the results of studies regarding the genetic basis of musicality (based on abilities such as musical perception, rhythm, or creativity) and studies based on extreme phenotypes associated with music (such as absolute pitch [AP] or synesthesia). Section 4 instead, reviews gene expression studies focused on the impact that music produces in the human genome, according to music engagement (listening and playing music). Next, in Section 5, we propose a new term, namely, sensogenomics, to refer to the study of the response of the genome to sensory stimuli. This concept is presented in reference to music, as one such powerful stimulus. Finally, the article also outlines the limitations of previous studies (Section 6), as well as the contributions of other fields, especially neurosciences and social sciences (detailed in Supplementary Data).

## 2. Methodology

Indexed searches were performed in PubMed using the following keywords: (i) “music AND neurol*” and (ii) “music AND genes”. The neurological search yielded 240 studies labelled as review articles, and they were published between 2010 and 2020 (the search was restricted to the last 10 years, due to the large amount of literature available; *n* = 2,360), of which 189 articles were retained (after filtering out irrelevant articles that fell outside the topic). The main topics covered by these 189 studies were: neurological bases of human musicality (*n* = 38), psychophysiology and the impact of music (*n* = 18), music as treatment and neurorehabilitation (*n* = 83), and general reviews (*n* = 3). A total of 156 studies could be retrieved, regarding genetics, but only 53 were finally retained as relevant after examining all the abstracts. Excluded papers were from other areas of research (biochemistry, physics, etc) or used the word “music” as a metaphor (e.g., musical chair games, software). Among the 53 studies retained, the main topics were on: candidate genes (*n* = 9), genetic expression (*n* = 7), music and diseases (*n* = 13), AP (*n* = 5), and general reviews (*n* = 7). Additional resources were explored by scrutinizing reference lists of the selected articles to identify additional studies not captured in the initial search. We also reviewed all the articles cited by the seven general reviews of genetics related to music published in the last ten years.

## 3. The Genetic Basis of Music in Human Evolution

### 3.1. The Origin of Musical Roots

The prehistoric mammoth ivory flutes, discovered in 2008 and belonging to the Aurignacian culture (dating to the Upper Paleolithic period), are among the earliest records of human technological and artistic innovations [11]. These flutes demonstrate the existence of a well-established musical tradition at the time when anatomically modern humans colonized Europe (>35,000 years ago); early humans were, therefore, making sounds even earlier than previously thought.

From anthropology and ethnomusicology, the historical and cross-cultural ubiquity of music is widely recognized [12,13], as well as its value in the culture and society of any civilization. The universality of musical behavior has also been widely noted: music appears in all traditional cultures, linked to religion, celebration, and dance [14].

New approaches trying to establish a dialogue between musicology and biology have used the term “Biomusicology” to designate the discipline concerned with the evolutionary history and biological roots of music, with different branches, such as evolutionary musicology or neuromusicology [15]. The field of the neuroscience of music has developed many arguments based on scientific research into musical brains, plasticity evidence produced when listening to and performing music, and especially, the possibilities of music as a therapeutic tool in certain diseases and disorders (see Supplementary Data).

How can we explain individual differences in ‘innate’ ability? In the most recent literature, genomic research emerges as a fundamental approach to understanding the biological bases of musical abilities. It seems evident that some genetic conditioning underlies musical abilities and musical perception, given the presence and importance of music in any culture [10] and our longstanding ancestral relationship (co-evolution?) with it. At the same time, it has long been debated whether musical talent is related to genes (“nature”) or to training and environmental stimulus (“nurture”). Although the importance of musical training, education, and environment in the expression and externalization of talent are not disputed, today there is ample evidence that points to a biological basis in musical talent and the perception of musical stimuli.

### 3.2. Genetic Basis of Musical Abilities

There is an incipient field of research focusing on the genetic bases that underlie musical ability [10], musical creativity [16], or AP [17,18,19].

Our biological connection with music does not seem different from other common biological conditions and disorders: while musicality most likely requires a given genetic background, an environmental component is needed to activate its development and progression. Musicality is considered the clear manifestation of musical abilities. Music education or teaching–learning experiences might be, in this sense, the true trigger. For example, a person will develop their potential as a musician if adequate musical training is superimposed on a given biological predisposition. Recent twin studies contributed to this debate with relevant arguments. Manzano and Ullen [20] studied highly discordant twins for piano practice, demonstrating the importance of brain development based on musical training. Mosing and Ullen [21] suggested the importance of genetic influence in the choice of the type of music and musical instrument, comparing monozygotic with dizygotic twins.

The following section firstly focuses on genes associated with different musical traits in the general population and secondly on the association of music with extreme phenotypes.

### 3.3. Genomic Background Associated with Musical Traits

The few studies available so far point to the existence of certain chromosomal regions, as well as family linkage, related to the way in which we perceive, process, or produce music. From a technical point of view, while the initial contributions were based on behavioral studies (familiar aggregation studies, twin studies, segregation analysis, and pedigree study), the most recent studies were more focused on molecular genetic approaches, such as genome-wide linkage studies (GWLS), genome-wide association studies (GWAS), exome sequencing, or copy number variation (CNVs) (see Table 1 for a more comprehensive summary).

Also, in the last decade, the interest in this topic has led to some review articles. Tan et al. [10] carried out the first comprehensive review of the genetic basis of musical ability, including an overview of human genetic methods (behavioral and molecular genetic approaches) and a summary of findings regarding music perception and music production abilities. Gingras et al. [9] presented a synthesis of literature regarding musicality at the extremes (amusia, AP, and altered musicality in genetic syndromes), as well as genome-wide linkage and association studies of musicality in the general population. The Department of Medical Genetics at the University of Helsinki has made a special contribution to the field, with several genomic studies on musical aptitude, perception, and practice [5,22,23], while Szyfter and Witt [24] recently presented a review study on genetics and musicality. The last review [25] introduced a stimulating narrative regarding the biocultural origin of music and its evolutionary function.

The next sub-sections summarize the results of previous studies related to genes that have been proposed to be associated with musicality.

#### 3.3.1. General Musical Abilities

Few genetic studies have investigated the biological basis of musical abilities. Two twin studies were pioneering in analyzing the heritability of general musical skills, although without a clear focus on the phenotype or an objective assessment of musical abilities [26,27]. According to Vinkhuyzen et al. [27], genetic factors are essential for outstanding levels of ability; Coon and Carey [26] found high correlations in monozygotic and dizygotic twins, also highlighting the important effect of a common environment.

Molecular studies have associated chromosomic regions and genes with musical abilities, indicating that specific loci or chromosomal regions are involved in different musical abilities, therefore suggesting a biological connection to musical roots. In this sense, chromosome 8q is involved in more than one musical characteristic [9]. The loci 8q21 and 8q24 seem to be related to AP [9,28] and 4q21–24 with musical perception [29,30], while the neighboring loci 4q23 and *UGT8* could be related to pitch precision when singing [31].

Besides, there is a recurrent association of *AVPR1A* gene, located on chromosome 12q, with different musical abilities: musical learning [32], musical perception [33], and musical memory [34,35]; and the *SLC6A4* gene has been found to be associated with musical memory [35] and choral participation [36]. The most recent analysis by Szyfter and Witt [25], also supports that the strongest statistical association between genes and musicality was provided for genes *AVPR1*, *SLC6A4*, *GALM*, *PCDH7*, and *GATA2*, as well as a few chromosome bands, such as 8q13–21, 4q22, and 4q23.

Consistently, a previous metanalysis, based on 105 molecular studies carried out on humans and other animal species [24], found a top candidate related to music abilities in humans in chromosome 4q21–q24; this region contains seven top candidate genes, namely, *MAPK10*, *SNCA*, *ARHGAP24*, *TET2*, *UBE2D3*, *FAM13A*, and *NUDT9*.

Finally, Järvelä [5] indicated that the *SNCA* gene was overexpressed when listening to and performing music, linking DNA- and RNA-based studies of music-related traits and dopamine metabolism.

#### 3.3.2. Music Perception

One of the pillars of musical perception is the ability to recognize or discriminate the pitch. One measure of pitch recognition utilized for several studies [20] is the distorted tunes test, which presents popular melodies containing some notes with incorrect pitch. Other studies have based the evaluation of pitch discrimination in other validated tools, as the Karma music test for auditory structuring ability and the Seashores test for pitch (SP) and time (ST) [16,29,30,32,33].

The first genetic study of pitch perception [20] explored the correlation existing in monozygotic and dizygotic twins, in relation to their abilities to detect discordant notes in popular melodies. This study revealed that the correlation is much greater in monozygotic than in dizygotic twins, supporting an additive genetic model, with an estimated heritability of 0.71 to 0.80. In contrast, a later pedigree study [29] found significant heritability but with a different percentage, according to the musical test utilized (for example, Karma music test [42%] and Seashore test for pitch [57%]).

Lately, molecular studies have begun to search for associations between genes and musical perception, and some statistically significant associations could be proposed. Ukkola et al. [33] found gene *AVPR1A* to be related to music perception and creativity, and adding confirmatory evidence in a posterior study [32]. Additionally, a new candidate gene for music perception was detected in a robust study with genome-wide copy number variation analysis (CNVs). Ukkola-Vuoti et al. [16] reported a deletion at 5q31.1 covering the protocadherin—a gene cluster (*PCDHA* 1-9) co-segregating with low music test scores (this gene cluster is involved in neural migration, differentiation, and synaptogenesis). The most recent molecular study on musical perception abilities [30], a GWLS-GWAS study, found an association with 4q21.23–22.1 and 4q24, which agreed with a preliminary study (chromosome 4q22) [29]. Additionally, three genes involved in inner ear development, *GATA2*, *PCDH7*, and *PCDH15*, were found to be strongly associated with musical aptitude.

Finally, Seesjarvi et al. [37] carried out an interesting twin study with controversial results. The method was based on three online music tests of musical perception, which showed no correlation. The first test, the scale test, aimed to detect pitch changes in a two-scale comparison and presented an important additive genetic effect (0.50), in contrast to the low rates for the environmental effect (0.07). In the second test, also based on pitch recognition but focusing on tonalities, the “out of key test”, surprisingly, presented the opposite pattern (0.58 for environmental effect and 0.03 for genetic effect), confirming the complexity of musical traits and the importance of music education as predictor of out of key. In a third test, related to rhythm perception, the authors reached conclusions in line with those obtained for the second test.

#### 3.3.3. Musical Memory

The genetic background of musical memory is an unexplored field, even the characterization of this kind of ability is not well identified. The neurobiology of musical cognition is an under-researched area and the musical memory is a sophisticated mechanism that remains poorly understood [38].

One study focusing on the genetics of musical memory [35] used validated musical memory measures (Gordon’s musical aptitude test and the tonal memory test for Seashore musical talents, among others) and the main results associated *AVPR1A* and *SLC6A4* with musical working memory. Even though this candidate gene study had a limited sample size, posterior studies replicated these findings (*n* = 343; 19 Finnish families), observing a strong association between memory for music and *AVPR1A* [33]. In the same vein, Israel et al. [39] suggested the role of *AVPR1A* and *OXTR* in musical abilities, such as dance and musical memory. Granot et al. [34] pointed out a pattern, whereby *AVP* modulates musical working memory throughout its ability to influence mood, attention, and arousal.

#### 3.3.4. Musical Creativity

Creativity in musical achievement is a complex trait that is manifested in different behaviors, such us improvisation, composition, and arrangements. The few genomic studies that tested this ability did so through a self-reported questionnaire [33,40], which constitutes an important limitation.

Firstly, musical creativity was the subject of a genetic study carried out by Ukkola et al. [33]. By analyzing 19 Finnish families, with at least some professional musicians and/or active amateurs, they discovered the following heritability results: 40% for composing, 46% for arranging, and 62% for improvising. Additionally, they found gene *AVPR1A* to be related to creativity. According to Donaldson and Young [41], this gene has been known to modulate social cognition and behavior, making it a strong candidate gene for music perception and production. These authors propose that music perception and creativity in music are linked to the same phenotypic spectrum of human cognitive social skills. Further, they noted that altruism and intense interest in music, together with relatively sparse language skills, are characteristic of Williams–Beuren syndrome (WBS); paradoxically, *AVPR1A* is also associated with autism spectrum disorder (ASD) [40], a phenotype characterized by poor social communication skills.

A few years later, Ukkola-Vuoti et al. [16] reported a deletion at 5q31.1 covering a gene cluster (*PCDHA* 1-9) co-segregating with low music test scores; this gene cluster is involved in neural migration, differentiation, and synaptogenesis. Creativity in music was also found to co-segregate with a duplication covering *GALM*, a gene related to serotonin. The limited power of the study (sample sizes: *n* = 170 experimental cases and *n* = 172 control subjects) and the difficult definition of the phenotype leads the authors to be cautious about the associations detected.

Finally, Oikkonen et al. [40] implemented a GWLS technique to 74 families (*n* = 474 individuals) and 103 unrelated subjects, founding evidence for linkage at 16p12.1–q12.1 for musical arranging and 4q22.1 for musical composition, lending further support to the 4q22.1 region as a candidate region for a broad range of music-related traits. They also proposed a common genetic background for music-related creative behavior and musical abilities at chromosome 4 and showed *CDK5* signaling with music listening and practice. Furthermore, they found the long-term depression pathway statistically over-represented in genes associated with composition.

#### 3.3.5. Musical Rhythm

Rhythm, understood as the synchronized movement regarding a musical beat, is a fundamental musical trait, although it is a practically unexplored field of research for genetics. The preprint by Niarchou et al. [42] was based on a GWAS carried out on a very large sample size (*n* = 606825) and analyzed volunteers, self-reporting their musical rhythm abilities. The participants were then tested through an internet-based phenotype for validation. Their results indicated that rhythm was associated with genes expressing in brain tissues and mapped to *VRK2*-*FANCL* as a top locus, indicating a biological connection between rhythm and neurodevelopment. This study found some replication of a gene near 4q22.1 (*CCSER1*) but did not find replication with many others candidate genes previously linked to musicality. These results may indicate a highly polygenic nature of rhythm as a musical trait.

#### 3.3.6. Musical Language Production: Singing

The ability to produce a specific vocal pitch is related to language, and it is, therefore, interesting to study the connection between music and language, as well as its materialization through singing. In connection with music, language requires the anatomical configuration of the vocal cords, larynx, pharynx, etc. A common evolutionary background of music and language has found support in recent studies, particularly ones that pointed out the importance of *FOXP2* in the processing of musical rhythm, language, and speech [17,43]. This highly conserved gene is only present in animals and it is related to the ability to vocalize. It is active in over 100 other genes, most of them involved in the development of the central nervous system. Campbell et al. [44] stated that Foxp2 protein is actively involved in musical language production in muroid rodents, being associated with a brain network responsible for fine motor production and auditory perception. However, the importance of *FOXP2* has been re-assessed in a recent study that considered the loss of function in our lineage [45], demonstrating the complexity of understanding its contribution to human evolution [46,47]. Therefore, language and musical language (understood as singing and pitch production through voice) could be developed as a highly complex trait.

The ability to produce a specific pitch is different to the skill of recognizing musical sounds and seems to be related to language and inner ear. A few studies have investigated this topic. Park et al. [30] carried out a complex study on 73 families in Mongolia (*n* = 1008), as part of the GENDISCAN study (gene discovery for complex traits in large, isolated families of Asians of the Northeast). The authors combined GWLS, GWAS, exome sequencing, and array-based comparative genomic hybridization (*ACGH*) for genomic analysis and a pitch-production accuracy test to determine this musical ability. Among its most important findings was the detection of a linkage peak in the 4q23 region, as well as a candidate region centered on the *UGT8* gene (4q26) for pitch production ability.

It may be argued that the ability to sing in a choir might be related to pitch production ability. In this vein, we may consider the contribution of Morley et al. [36], despite its limitations, namely that they did not use a formal music test to evaluate the ability of pitch production and that they enrolled 262 amateur singers from nine different choirs and 261 controls without making any correction for ethnicity/ancestry (population stratification). The main result of this candidate gene study was the association of *STin2* VNTR, in the *SLC6A4* gene, with choir participation.

### 3.4. Genetics and Music in Extreme Phenotypes

The present section deals with the association of music with extreme phenotypes for which there is convincing genetic evidence. There are other phenotypes for which there exists emerging evidence from neurology and neuroscience but lack studies in the field of genetics (e.g., amusia, see Supplementary Data).

#### 3.4.1. Genetic Research on Absolute Pitch (AP)

AP is an extreme phenotype characterized for the ability to recognize and/or produce a sound with a specific pitch without reference. Baharloo et al. [18] indicated that although there is no evidence for the underlying developmental mechanisms that may play a role in the AP phenotype, early musical training is not sufficient for its development. These and other authors state that, while AP must be at least in part inherited, environment plays an important role, such that early childhood exposure or musical training is most likely the single most important factor for the development of AP [18,48,49].

Some early studies pointed to the existence of a genetic basis for AP. For example, the segregation study, conducted by Profita and Bidder [50], showed pedigrees with a high prevalence of AP. The segregation rate observed by these authors was 0.24–0.37, suggesting AP is associated with an autosomal dominant characteristic with incomplete penetrance. Although there is no clear consensus on the prevalence of AP, an indicative figure would be 1 in 10,000 [50,51,52]. Among musicians, the proportion of AP is much higher, 8.8% according to Wellek [53], and 3.4% according to Revesz [54].

In a study of carriers, Baharloo et al. [18] provided evidence that individuals with AP were four times more likely to report other members of their family with AP than those who did not have AP. Regardless of the musical training undergone by a person or developed in the family environment, the family aggregation detected in this study clearly suggested the existence of genetic factors underlying the development of AP, with a sibling recurrence risk of 7.5. Other studies of familiar aggregation reported similar values for sibling recurrence risk: 8.3 [55], 7.8–15.1 [56], and 12.2 [57].

The observation that AP is more common among speakers of tonal languages, such as certain dialects of Chinese (e.g., Mandarin and Cantonese) or Vietnamese, points in the same direction. In these languages, the meaning of the lexicon strongly depends on the pitch of the sound. This fact would indicate the existence of genetic factors, with a higher prevalence in certain population groups and, to a certain extent, point to a co-evolution of cultural factors as important as language with the biological capacities to interpret it, in a kind of positive selective evolution. Thus, for instance, AP is significantly more prevalent in Asian students, compared to all other non-Asian ethnic groups [55].

A genome-wide study of 73 families with AP [28] reveals a strongest linkage for 8q24.21 and locus heterogeneity (locus 21.11 and genes *GSDMC*, *FAM49B*, *ASAP1*, and *ADCY8*) but with inconsistent findings; the authors concluded that AP is probably genetically heterogeneous. In agreement with these results, the family segregation analysis and twin study by Theusch et al. [28] claimed heterogeneity of AP, based on a segregation ratio (0.089) and the concordance rate for monozygotic (78.6%) and dizygotic twins (45.2%).

A few reports have indicated a relationship between AP and other clinical phenotypes, such as congenital blindness [56], WBS [58], ASD [59,60,61], and synesthesia [49,62].

#### 3.4.2. Biological Bases of Sound-Color Synesthesia

Synesthesia is a rare, nonpathological phenomenon where stimulation of one sense automatically provokes a secondary perception in another [63]. It is rare in the general population, with a prevalence rate of approximately 1 case in 2000 [64]. Interestingly, it appears to exhibit strong familial aggregation [63,64].

The hereditary nature of synesthesia is widely accepted. Studies to date suggest that the genes involved are related to mechanisms of cortical connectivity and that synesthesia is an oligogenic phenomenon subject to multiple modes of inheritance. However, the specific genes that contribute to its development are still unknown. It is also unknown whether the genetic bases of the different modalities of the phenomenon are common or idiosyncratic for each of them [65]. To the best of our knowledge, there are three relevant genetic studies on synesthesia.

First, the Cambridge Synesthesia Research Group suggests that sound-color synesthesia is an oligogenic phenomenon subject to multiple modes of inheritance and heterogeneously localized [66]. These authors carried out a whole genome scan and fine-mapping linkage study of 43 multiplex families, including several subjects who had sound-color synesthesia, finding association with different chromosome locations (2q24, 5q33, 6p12, and 12p12) related to cortical connectivity.

In the second study, three families with sound–color synesthesia, affecting multiple relatives across various generations (included as part of the previous study), were selected for a whole-exome sequencing (WES) by the same research group. They identified rare genetic variants co-segregating with synesthesia in each family, uncovering 37 genes of interest. Six genes, *COL4A1*, *ITGA2*, *MYO10*, *ROBO3*, *SLC9A6*, and *SLIT2*, were particularly relevant because they are associated with axonogenesis and express during infancy when synesthetic associations are supposed to be shaped [63].

Finally, Gregersen et al. [49,62] established a relevance relationship between AP and synesthesia. These authors carried out a GWLS on 768 AP subjects and, under a dominant model, they found that the 20.1% of AP cases reported synesthesia, especially related to sound-color. Despite the failure to replicate AP association from prior studies, these authors observed a good candidate region on chromosome 6q14–q16 (which contains several genes involved in neurodevelopment), as well as evidence of linkage in chromosome 2q22–q24, indicating a genetic relationship between AP and synesthesia.

#### 3.4.3. Williams Syndrome and Music

Williams syndrome (also known as Williams-Beuren syndrome, WBS) is a genetic disease that affects about 1 in 20,000 newborns [67]. This condition is characterized by mild to moderate intellectual disability or learning problems, unique personality characteristics, distinctive facial features, and heart and cardiovascular disorders. Patients with WBS are recognized by their great musical ability [68]. However, the functional and genetic basis of this unusual auditory phenotype is currently unknown. To a certain extent, this ability appears in the form of hyperacusis, defined as an extreme sensitivity to sound that can become annoying and painful.

Examining the neural basis of auditory processing of music and noise in WBS patients (through functional magnetic resonance imaging) researchers found evidence of a different neurofunctional organization from that in normal people [69]. WBS presents a relative enlargement of the left temporal plane, also characteristic of musicians with AP [58].

Levitin [68] reviewed a series of studies of the musical abilities and behaviors of individuals with WBS, remarking the increased activation in the right amygdala to music and to noise and concluding that “Williams syndrome represents a compelling model of the relationship between genes, brains, and such complex cognitive behaviors as music”. According to a most recent research, WBS individuals show extreme and almost exclusive holistic sound perception, similar to that reported by professional musicians [70].

In the field of genetics, Canales et al. [71] investigated the involvement of *GTF2IRD1*, a transcription factor encoded by a gene located within the WBS deletion that has been implicated as a contributor to the WBS assorted neurocognitive profile and craniofacial abnormalities. This work suggests that *GTF2IRD1* insufficiency is not enough to explain all the hypoacusis. Several genomic locations have been associated with WBS, e.g., a microdeletion and a microduplication of approximately 1 to 3 Mb in chromosome 7 (7q11.23) [72]. The chromosome is flanked by short repeats of sequences known as LCRs (Low Copy Repeats) that have a sequence identity (>97%). In most cases the mutation is not inherited from the parents but occurs de novo. Relevant research revealed significantly higher median levels of oxytocin (*OT*) and arginine vasopressin (*AVP*) in WBS subjects *versus* controls at baseline, with a less marked increase in *AVP* [73]. Besides, *OT* and *AVP* increased in response to music in WBS subjects, with greater variability and an amplified peak release, compared to controls.

#### 3.4.4. Autism Spectrum Disorder (ASD) and Music

Patients with ASD suffer from a developmental neurological disorder, which manifests in difficulties to communicate, repetition of phrases (echolalia), and repetitive movements (stereotypies). Not all patients display the same symptoms in the same ways, hence the talk of the autism spectrum, rather than a single condition of ASD.

It is widely reported that many people with ASD have a special sensitivity to music and an ability to distinguish a greater range of tones [74]. Patients with ASD generally have different sensory experiences, with musical perception occupying a prominent place among their abilities [61]. As a form of nonverbal communication, music is a powerful and accessible affective stimulus that captures and emotionally rewards individuals with ASD [75]. Most ASD patients have a great melodic memory, which allows them to recall entire melodies with great ease [61]; and AP is considered more common in patients with ASD, although the true prevalence has not been established with any degree of accuracy.

Molecular genetic studies in human behavior have highlighted the role of the arginine vasopressin 1a receptor (*AVPR1a*) and the oxytocin receptor (*OXTR*) in connection with musical abilities, such as dance and musical memory [39], and according to several studies, genetic variants in these two neuropeptides could potentially contribute to an increased risk of ASD [76,77,78].

#### 3.4.5. Prader-Willi Syndrome, Genomic Imprinting, and Music

A recent investigation on Prader–Willi syndrome (PWS), a rare disorder related to genomic imprinting (genes from chromosome 15q11–q13 that are typically paternally expressed are unexpressed), proposed PWS as a distinctive musical phenotype, informing theories of music’s evolutionary history [79]. These researchers reported unusual responses to music in people with PWS: they moved more during music listening, exhibited greater reductions in heart rate in response to music listening, and displayed a specific deficit in pitch-discrimination ability; from this, they concluded that paternally expressed genes from 15q11–q13, which are unexpressed in PWS, may increase demands for music and enhance perceptual sensitivity to music.

## 4. The Impact of Musical Stimuli in Our Genome

Many investigations in different fields have argued for the effects of music on the intellectual, social and personal development of human beings [80]. However, ancient precedents of this approach could be mainly found in a distant discipline: the aesthetics of music, with philosophers advocating the power of music for human transformation (see Supplementary Data).

Nowadays, in the field of neurosciences, there is a growing interest in studying the impact of music in our brain, to explore the functioning of the brain itself, arguing that playing, listening to, and creating music involve practically every cognitive function [1]. This discipline has focused their advances in how music activates and interconnects brain areas, developing brain plasticity. Other studies have explored the influence of environmental-cultural and biological factors on musical perception, as well as the conditioning of musical training on the brain responses to music.

Also, the field of psychophysiology of music has studied the biological role of music and its therapeutic role in diseases and disorders, such as ASD, Alzheimer, motor rehabilitation, Parkinson, or epilepsy. More generally, researchers have studied the impact of music related to neural bases of emotions, as a trigger of changes in heart rate, respiration, blood pressure, skin temperature and conductivity, muscle tension and biochemical responses; its impact in reduction of stress, or in the immune system (see Supplementary Data).

### Genomics and Musical Stimuli: Impact of Music in Gene Expression

The study of the impact of music in gene expression remains an under-research area. Bittman et al. [81], can be considered pioneers in this field; analyzing the expression of a few immune response-related genes using qRT-PCR, they showed that recreational music-making has the ability to modulate the human stress response. Additionally, Qu et al. [82] carried out a gene expression analysis collecting peripheral blood samples before and after being exposed to a 60 min session of classical or relaxing jazz music; however, the main goal of this study was to analyze the results of a comprehensive yoga program, with music listening as a control activity.

Focusing on the effects of musical stimuli on gene expression, only a few authors have studied the effect of music on the transcriptome. Kanduri et al. [83] investigated the effects of music listening on the public transcriptome after a 20-min classical music concert of Mozart’s Violin Concerto No. 3 in G major. Comparing the transcriptional responses of music listening for musically experienced and inexperienced participants, significant transcriptional responses were observed only in individuals musically trained or with high musical aptitude scores. This suggests that certain musical abilities (whether innate or acquired) may influence the transcriptional responses of listening to music.

This research revealed that the up-regulated genes are known to be associated with dopamine signaling, synaptic neurotransmission, synaptic function, learning, memory and cognitive performance, song learning and singing in songbirds, auditory cortical activation, AP, neuroprotection, and neurogenesis. One of the most up-regulated genes, *SNCA*, is in the best candidate linkage region of musical aptitude on chromosome 4q22.1 and is regulated by *GATA2*, which is known to be associated with musical aptitude. On the other hand, down-regulated genes are known to cause mammalian neuronal apoptosis, immoderate oxidative phosphorylation, and deficits in dopaminergic neurotransmission, which are the characteristics of neurodegeneration.

The same experimental concert served to study the effects of music-listening on gene regulation by sequencing microRNAs of the audience [84]. The identified microRNAs were shown to affect dopamine metabolism and to prevent neurodegeneration. They identified up-regulation of microRNAs responsive to neuronal activity (miR-132, miR-23a, and miR-23b); these microRNAs are implicated in neuronal plasticity and are responsible of cognitive functions related to memory; it is worth noting the function of miR-23a in long-term memory and the role of miR-132 and *DICER* in dopaminergic pathways. According to previous research, *FOS*, *CREB1*, *JUN*, *EGR1*, and *BDNF*, as transcriptional regulators of the up-regulated microRNAs, have been associated with musical qualities. Other genes associated with musical aptitude, such as *GATA2*, which regulates the expression of *BDNF* and *SNCA*, co-expressed and up-regulated in music listening and performance.

In another similar experimental design aimed at studying the transcriptional response to musical performance, Kanduri et al. [85] analyzed the response of professional musicians after playing music. They analyzed the transcriptional responses after a 2-hour concert performance and after a ‘music-free’ control session, in peripheral blood samples. Among the results obtained are certain groups of genes that were over-expressed, such as those related to dopaminergic neurotransmission, motor behavior, neuronal plasticity, and neurocognitive functions, such as learning and memory. Prominent genes include *SNCA*, *FOS*, and *DUSP1*; curiously, these genes are involved in the perception and production of sound in birds, which, according to the authors, suggests that there are well-conserved evolutionary pathways in the biological processes related to music. Additionally, the modulation of genes related to calcium ion homeostasis, iron ion homeostasis, glutathione metabolism, and several neuropsychiatric and neurodegenerative diseases implies that music performance may affect the biological pathways that are otherwise essential for the proper maintenance of neuronal function and survival. Thus, Kanduri et al. [85] stated that genes related to the neuropsychiatric and neurodegenerative disorders of music performance may partially explain the effect of music as a therapeutic tool in clinical settings.

The same research group analyzed the performance of professional musicians, with a special focus on how classical music affects their microRNA expression after two hours playing music [86]. Their aim was to investigate a putative gene regulatory network representing the molecular mechanisms underlying music performance in professional musicians. Despite their limited sample size, they found two microRNAs up-regulated after music performance, namely hsa-miR-92a-3p and hsa-miR-222-3p, which target *FOXP2* and constitute a microRNA-*FOXP2* gene regulatory network. According to these authors, some of the molecules in the network were important for cell survival, cellular proliferation, neuron survival, long-term potentiation, and dopamine signaling; some of the molecules, such as *GATA2*, *GATA3*, and miR-222, have functions in the inner ear development.

Recently, a pioneer study by Ramirez-Rivera et al. [87] aimed at evaluating the effect of music on the proliferation and gene expression in gastric cancer cells (AGS). Cells were cultured while exposed to one of two musical genres: classical (Ludwig Van Beethoven) or death metal (Cannibal Corpse) for 12 h nonstop. They noted an increase in the proliferation of AGS gastric cancer cells exposed to death metal music, which was not observed with classical music treatment. This suggests that certain types of music can alter the cell cycle in cancer cells. The gene expressions of *CASP3*, *CASP8*, and *CCNB1* increased in response to both musical genres (especially in the cells subjected to classical music treatment) and their up-regulation in gastric cancer is associated with less aggressive tumor behavior, which could indicate that music treatment causes cancer cells to act less aggressively. Additionally, a strong down-regulation of two genes was noted: *TP53* in cells exposed to classical music and *PUMA* in cells exposed to death metal. According to the authors, gene expression can be altered by the frequency and amplitude of soundwaves, although the authors could not provide insights into the mechanisms acting behind this phenomenon.

All these findings could be of interest to advance on the potential use of music as a therapy. Despite the important limitations of these studies (see bellow), especially related to the limited sample sizes, the genetic response to musical stimuli appears to be a promising research field. Based on the evidence regarding the impact of musical stimuli in our genome, in the following section, we develop a proposal.

## 5. Sensogenomics and the Genetic Response to Musical Stimuli

Music is rewarding, motivating, and one of the most valued of all human cultural accomplishments. The processing and perception of music in healthy and diseased individuals constitutes an exciting area to study the impact of music as human beings. The uniqueness of our human interest in music from the early days of life suggests the existence of a biological background (most likely connected with gene variation and gene expression) triggered by musical stimuli.

The main premise behind sensogenomics is that human knowledge begins through sensation and perception. The present article introduces the field of musical sensogenomics, focusing on the impact of musical stimuli as a powerful trigger. Sensogenomics could be defined as a research area that investigates the mechanisms and potential of the sensory activation of genes. How do sensory stimuli affect the genome? What happens to RNA expression when confronted with these stimuli, in health and in disease? Following from the above definition, musical sensogenomics implies that being involved in musical activities or simply listening to music produces more than pleasure, but where does the emotional power of music come from?

The sensation of hearing occurs when the waves that produce vibrations in the air are collected in the outer ear and transmitted through the inner ear to the auditory nerve [88]. According to neuroscientists, a sound reaching the eardrum sets in motion a complex cascade of mechanical, chemical, and neural events in the cochlea, brain stem, midbrain nuclei, and cortex that eventually (but rapidly) results in a percept [89].

With the arrival of parallel sequencing techniques and the relatively young ‘-omic’ era, more effort should be devoted to the biogenetic study of the ways music can impact the expression of our genome, in a new discipline we propose to call sensogenomics. A retrospective look at the literature indicates that the new techniques in genomics have hardly been used in the study of musical skills; the advent of molecular genetics holds much promise for this relatively underexplored field. We contend that the empirical studies of gene expression, under well-controlled experimental conditions with statistical power, could help us understand the role of music in human biology and different pathological conditions. This might, in the long-term, assist in the development of proper therapeutical interventions.

Genomics enables us to study biological phenomena without any knowledge of the biological background of the phenotype. The success of genomics has been demonstrated in medical research, where thousands of genes that cause inherited diseases or predisposition to multifactorial disorders have been identified. Further research is needed to explore these genomic bases of music stimuli in an ‘orchestrated’ field of scientific research that integrates, among others, genomic, epigenomics, and gene expression, while paying attention to neurological discoveries. This will give rise to a new era of sensogenomics.

## 6. Limitations of Present-Day Studies

There are a number of limitations in the studies carried out until now. Most of which are however shared with other fields of biomedical research.

First, the phenotypic characterization of the individuals under study is complex [90]. Some measures used to establish the phenotypes are not always fully validated or are based, at least partially, on self-reported assessment of musical abilities [16,26,27,42,55,62]. It should be considered that, even in the fields of music education and psychology of music, there is no agreement on the definition of musicality or musical abilities, with many different musical tests used to measure these features. Even for the most popular extreme phenotypes, e.g., AP or sound-color synesthesia, there are debated issues among specialists. Second, earlier behavioral genetic investigations of music ability suffered from questionable methodologies [10] (i.e., the lack of adequate control groups or issues with under-reporting), especially in studies of music therapy. Third, typically association studies of musical skills are based on limited sample sizes, compared with GWAS studies in other complex genetic traits, and so have been relatively underpowered [30,31]. Gene expression studies aimed at analyzing music listening or performance also have limited sample sizes [81,82,83]. Moreover, studies based on candidate genes have a limited replication rate [91]. This could explain why some recent genome-wide screens failed to confirm candidate genes from prior studies. For example, the genome-wide linkage and association study by Oikkonen et al. [30] did not replicate the previous association with the region pinpointed for AP and other associations near the *AVPR1A* gene. According to Gingras et al. [9]: “Although linkage and association are different types of tests, it is unusual that there were no genetic markers showing convergent evidence from both methods”. In this regard, Hewitt [92] claimed that there are only a few rigorous studies including proper validation. In this vein, Morley et al. [36] pointed out that the lack of correction for ethnic homogeneity in the association analysis could cause false positive results in candidate gene studies.

## 7. Conclusions

**Figure 1:**
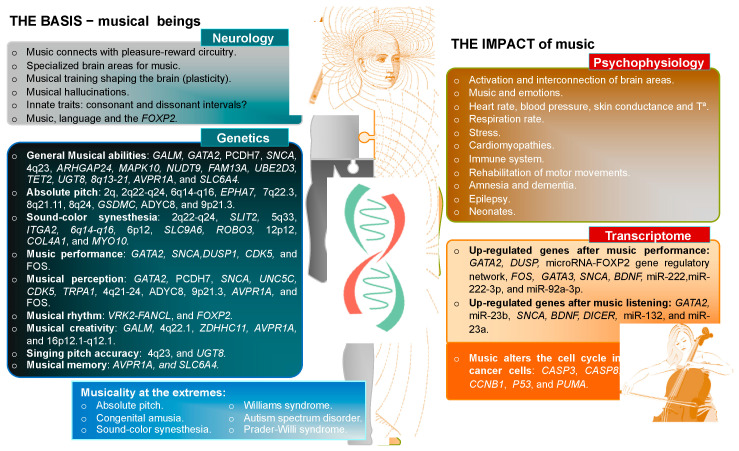
Scheme summarizing the known genetic and neurological basis of music and its impact on psychophysiology and transcriptome, according to the state-of-the-art of science.

Despite the limitations, the present article has reviewed many studies that convergently support strong evidence for a biological, highly polygenic background connected to music. Complementary approaches from social sciences, neurosciences, psychophysiology, and genetics have demonstrated the importance of nature and nurture in our characterization as musical beings (Figure 1). The arguments for the biological bases of musicality, as well as the evidence of the powerful stimulus that music represent to our genome, brain, and psychophysiology, are sufficiently robust to warrant a promising future for studies on this subject.

The close relationship between biology and music seems to have co-evolved with anatomically modern humans from Paleolithic times to present. There is widespread scientific agreement that this biological background becomes clearly visible when it shows up in the form of unique musicians and their abilities to compose or interpret music (but also in several biomedical disorders). Future interdisciplinary studies, inspiring connection between fields and findings, as well as expanding opportunities for studies of music perception [93] and beyond, are needed to shed light on this evolutionary connection between music and biology. This review has sought to highlight the potential of an under-researched area: the study of the impact of musical stimuli in our genome, a new perspective on ‘sensogenomics’.

## Figures and Tables

**Table 1:** Experimental genetic studies related to music.

	STUDIES	FOCUS	TYPE OF STUDY	CASES/CONTROLS
DNA: MUSICAL TRAITS	Coon and Carey (1989)	Musical abilities	Twin study	*n* = 98 MZ and 70 DZ males + 93 MZ and 132 DZ females
	Vinkhuyzen et al., (2009)	Musical abilities	Twin study	*n* = 1685 twin pairs
	Drayna et al., (2001)	Music perception	Twin study	*n* = 136 MZ + 148 DZ
	Pulli et al., (2008)	Music perception	GWLS/pedigree study	*n* = 234 from 15 families
	Seeajärvi et al., (2016)	Music perception	Twin study	*n* = 384 (69MZ, 44DZ + 70MZ, and 88DZ, without a co-twin)
	Ukkola-Vuoti et al., (2009)	Music perception/creativity	Candidate genes/pedigree study	*n* = 343 from 19 families
	Ukkola-Vuoti et al., (2011)	Music perception	Candidate genes	*n* = 437 from 31 families
	Ukkola-Vuoti et al., (2013)	Music perception/creativity	Genome-wide CNVs	*n* = 170 cases from 5 families + 172 controls
	Oikkonen et al., (2015)	Music perception	GWLS/GWAS	*n* = 767 from 76 families
	Oikkonen et al., (2016a)	Musical creativity	GWLS	*n* = 474 cases from 79 families + 103 controls
	Granot et al., (2007)	Musical memory	Candidate genes	*n* = 82 cases
	Park et al., (2012)	Musical language production	GWLS/GWAS/exome sequencing/aCGH	*n* = 1008 from 73 families
	Morley et al., (2012)	Musical language production	Candidate genes	*n* = 262 cases + 261 controls
	Niarchou et al., (2019)	Rhythm	GWAS	*n* = 606,825
DNA AND EXTRE PHENOTYPES	Profita and Bidder (1988)	AP	Family segregation analysis	*n* = 35 AP from 19 families
	Gregersen and Kumar (1996)	AP	Familiar aggregation	*n* = 101 AP
	Baharloo et al., (1998)	AP	Familiar aggregation	*n* = 612 (92 self-reported AP + 520 non-AP/controls)
	Gregersen et al., (1999)	AP	Familiar aggregation	*n* = 2707 music students/249 AP
	Baharloo et al., (2000)	AP	Familiar aggregation	*n* = 74 AP with 113 siblings + 625 controls
	Gregersen et al., (2001)	AP	Familiar aggregation	*n* = 1067 cases (Asian/no Asian)
	Theusch et al., (2009)	AP	GWLS	*n* = 281 from 73 AP families with at least 2 AP possessors
	Theusch and Gitschier (2011)	AP	Family segregation analysis/twin study	*n* = 1463 families + 14 MZ + 31 DZ
	Gregersen et al., (2013)	AP/synesthesia	GWLS/exome sequencing	*n* = 768 AP subjects/151 reported synesthesia
	Asher et al., (2009)	Sound-color synesthesia	Whole-genome scan/fine-mapping linkage study	*n* = 196 from 43 multiplex families
	Tilot et al., (2018)	Sound-color synesthesia	WES	*n* = 3 families
	Peretz et al., (2007)	Congenital amusia	Family aggregation study	*n* = 71 cases + 75 controls
	Kalmus and Fry (1980)	Congenital amusia	Pedigree study/twin–sibling study	*n* = 600
	Lai et al., (2001)	Speech and language disorder	Gene expression analysis/DNA sequencing	*n* = 3 generation pedigree
MUSIC AND GENE EXPRESSION	Bittman et al., (2005)	Music performance and stress	Gene expression analysis	*n* = 32 (16 cases + 16 control)
	QU et al., (2013)	Music listening and stress	Gene expression analysis	*n* = 14 cases
	Kanduri et al., (2015a)	Music listening	Gene expression analysis	*n* = 48 cases/audience + 15 controls
	Kanduri et al., (2015b)	Music performance	Gene expression analysis	*n* = 10 professional musicians + music-free control session
	Nair et al., (2020)	Music listening	Sequencing microRNAs	*n* = 48 cases/audience + 15 controls
	Nair et al., (2019)	Music performance	Sequencing microRNAs	*n* = 10 professional musicians + music-free control session
	Ramirez-Rivera and Bernal (2019)	Musical impact	Gene expression analysis	*n* = 5.000 gastric cancer cells

Note. Genome wide linkage study (GWLS); copy number variation analysis (CNVs); genome-wide association study (GWAS); array-based comparative genomic hybridization (aCGH); whole-exome sequencing (WES); monozygotic (MZ); dizygotic (DZ).

## Supplementary Materials

The following are available online at https://www.mdpi.com/article/10.3390/genes12091454/s1, Supplementary Data.
