# Supplementary material for: Sensogenomics and the Biological Background Underlying Musical Stimuli: Perspectives for a New Era of Musical Research
Source: Genes (Basel). 2021 Sep 21;12(9):1454. doi: 10.3390/genes12091454 (PMC8472585; doi:10.3390/genes12091454)
Supplement: Supplementary file 1 [file genes-12-01454-s001.zip › genes-1343034-supplementary.pdf]

SUPPLEMENTARY DATA

Sensogenomics and the Biological Background Underlying Musical Stimuli: Perspectives for a New Era on Musical Research

Laura Navarro, Federico Martinón-Torres, Antonio Salas

Neurobiological basis of musicality

The neuroscience of music has become an important research field, and several reviews have appeared in the last five years that aims at exploring the state of the art on the biological origins of music, brain adaptations, the therapeutic potential of music brain plasticity elicited by listening or performing music , and the beneficial effect of music listening and performing on individuals with brain disease and neurological disorders, among other [1-3].

The evolutionary basis of the connection between music and speech remains unresolved. Is the vocal communication in animals a symptom of music origin? According to Brown [4], music and language are “reciprocal specializations of a dual-natured referential emotive communicative precursor, whereby music emphasizes sound as emotive meaning and language emphasizes sound as referential meaning”. Comparative studies between musical abilities and language could help further understand the neurophysiological aspects common to these two abilities, as well as their evolutionary origins [5] and the molecular mechanisms that underlie the associated pathologies.

Many animal species use sounds intrinsic to their biology to communicate or perform other types of tasks necessary for their survival [6]; some can sing in specific keys with complex musical forms, remember songs, and even learn to sing. Songbirds and whales shows learned vocalizations even with large repertories [7]. Slater [8] registered around 4,000 songbirds with a rich variety of vocal pattering. Marler [9] indicates that mockingbirds can create hundreds of patterns (musical phrases) and to copy musical form and tempo from other species. Recent research shows that some bats are able to sing like birds and humans [10] showing vocal flexibility and vocal usage learning.

Many neuroscientists have claimed the existence of innate musical traits. Current neurophysiological studies have identified neural correlates of consonant and dissonant pitch relationships at the cortical level in humans. Fishman et al [11] studied the reaction of three macaques and their differential reaction to consonant and dissonant musical intervals, showing more brain activity with dissonance; according to these authors, the magnitude of oscillatory phase-locked activity in the auditory cortex correlates with the perceived dissonance of the musical chords. Thus, dissonant chords (e.g. minor and major second intervals), display oscillations phase-locked to the predicted difference frequencies, whereas responses evoked by consonant chords (e.g. octaves and perfect fifths), led to little or no phase-locked activity. The data were compared with the results of two people, the results suggesting that phase-locked oscillatory responses in monkey auditory cortex are not epiphenomena but likely represent an important component of the auditory cortical representation of sensory dissonance. Crespo-Bojorque et al. [12] discovered that consonant musical intervals tend to be more readily processed than dissonant intervals. Changes in a sequence of consonant intervals are rapidly processed independently of musical expertise, in both musicians and non-musicians. These authors also observed a facilitator effect of musical expertise at a neural level: participants with extended musical training showed automatic neural responses in both consonant and dissonant sequences, suggesting that experience allows for more efficient processing of dissonant intervals.

In this regard, research during the last decades has greatly increased our general understanding of brain plasticity, with wide implication for areas beyond neuroscience and for all human life. Music training causes structural changes in the brain. For example, string players show a larger representation in the motor cortex of the non-dominant hand [13]. In this line, Johansson [14] indicates that early musical training has lasting effects in shaping the brain.

Peretz [13] indicated that the brain has specialized areas whose main functions are musical processing, and which do not intervene in the processing of other types of information. This author maintains that there is a specific cognitive domain for music, and a sample of the biological bases of musicality would be the ability to process specific musical tones – something that is not necessary connected to any other type of biological function. The existence of

these areas suggests that human beings have an innate capacity for musical processing [15]. Furthermore, neurological studies have shown that musical abilities can utilize different processing modes, because the human ability to keep time and rhythmic development is dissociable from the capacity to produce and perceive the tonal features of music [16,17]. Merker et al. [18] also demonstrated that behavioral synchrony to a regular beat is a distinct feature of the human brain.

In the same vein, the existence of music-specific neural networks is found in various pathological conditions, based on brain damage or congenital brain anomalies that segregate musical abilities from the rest of the cognitive system [19]. Neuroscientists studying AP have reported an exaggerated leftward asymmetry in the planum temporale of AP subjects compared to controls matched by musical training – a finding that suggests a biological basis for this anatomic feature [20,21]. Elmer [22] found increased connectivity in the left but not in the right hemisphere in AP compared with non-AP musicians.

Research has also been made on musical (auditory) hallucinations, which are defined as false perceptions experienced in a waking state, and which are not a consequence of stimuli from the external environment; they can also involve any sensory modality [23]. Musical hallucinations can occasionally result from focal lesions in the brain and from psychiatric disorders, but they are also common in the non-psychiatric population. Musical hallucinations, often related to prior musical experiences, are considered a pathology on the border of psychiatry, neurology, and otorhinolaryngology, but not necessarily related to mental illness [24]. Among the famous musicians who may have suffered musical hallucinations are Joseph Haydn, Gaetano Donizetti or Maurice Ravel [25].

Finally, according to Blood & Zatorre [26] music connects with biologically relevant, survival-related stimuli via their common recruitment of brain circuitry involved in pleasure and reward. These authors claimed that music stimuli activate brain structures/networks (e.g. basal forebrain and certain brainstem nuclei, cortical areas involved in emotional evaluation), that are known to be active in response to other euphoria inducing stimuli (e.g. food, sex, and drugs of abuse).

Historical precedents from aesthetics of music

The evidence for music as a therapy in early history has been compiled by Thaut [27] in four broad historical–cultural divisions: preliterate cultures, early civilizations (Mesopotamia, Egypt, Israel), Greek Antiquity, and from Middle Ages to Baroque.

The Greek philosopher Aristides Quintilianus, bringing together Pythagorean, Platonic and Aristotelian thought, linked mathematics, philosophy, and music in his explanation of the world, which he captured in his famous treatise *On Music*. Here, he defended the power of music by establishing a system that links numbers and the cosmos. Likewise, he associated the four numbers of the tetraktýs: one to wisdom, two to courage and strength, three to moderation and beauty, and four to justice and health [28,29].

The theories about Ethos in the Pythagorean and Platonic tradition are most notable. Ethos represents the power that a sound can exert on the human being. It is an idea present throughout the entire Greek musical tradition of the Pythagorean and Platonic court: to attribute to specific musical instruments, as well as to certain rhythms and especially musical scales (modes) the power to modulate our mood and even our own moral, which Greek philosophers and theorists referred to as "the ethical power of music."

Greek ethos theory brings together many aspects of a belief held by various Hellenic authors and by some later figures; among them poets and philosophers of the greatest eminence, expressed in differing ways their belief that music can convey, foster and even generate ethical states [30]. Damo, a Pythagorean philosopher, was one of the most important figures in the development of the concept of musical ethos. Damo expanded and codified doctrines of ethos to a notable and perhaps unparalleled extent: his view that music is connected with the soul's motion provided one of the main theoretical foundations on which Plato built his own thoughts; and his name enjoyed wide recognition until the Roman period and even later (Cicero, On Oratory, iii.33; Martianus Capella, ix.926)[31].

According to Aristotle’s theories of psychology and perception, the actual experience of music should not be considered in ethical terms: musical experience is not an attitude of the soul but merely a pathos, something that

happens to one [30]. These theories on the relationships between ethos and the power of music will be widely developed during Antiquity by some of the most important Greek philosophers and will have wide repercussions in the Roman and medieval world.

These ideas are recovered in the Baroque, giving rise to the "Theory of affections" or "Doctrine of affections". Throughout the Baroque period, the Doctrine of affections governed musical composition through the musical elements of intervals, key, and tempo because music is capable of arousing affections within the listener to produce an intended emotional response. At the center of the doctrine was the belief that, by making use of the proper standard musical procedure or device, the composer could create a piece of music capable of triggering specific, involuntary emotional responses in the audience. These devices and their affective counterparts were rigorously cataloged and described by such 17th-18th century theorists as Johann Mattheson (1681-1764) who was especially comprehensive in his treatment of the affections in music: for example, he noted that joy is elicited by large intervals, sadness by small intervals and fury may be aroused by a roughness of harmony coupled with a rapid melody [32].

Neurosciences and the musical stimuli

Neuroscientists have used music to advance our knowledge about speech, brain plasticity and even the origins of emotion; according to Zatorre and McGill [33] “art and culture must have their origins in the function and structure of the human nervous system”. Listening, performing, or e.g., musical improvisation are attracting growing interest among the scientific community, since they are considered among the most complex activities from the cognitive point of view. It has been demonstrated that these musical activities have the power to activate brain areas, as well as the complex interconnection of others [34,35]. Even a seemingly simple activity, such as humming a familiar tune, necessitates complex auditory pattern-processing mechanisms, attention, memory storage and retrieval, motor programming, sensory–motor integration, and so forth.

Technological advances, including functional magnetic resonance imaging (MRI / fMRI and EEG) allow knowing which areas of the brain are activated, deactivated, or connected. The availability of modern neuroimaging techniques such as functional magnetic resonance imaging, positron emission tomography

(PET), neurophysiology (magnetoencephalography) and the introduction of more refined psychological paradigms are providing additional information on how brain processes produce music [36].

However, there are noteworthy weaknesses in many neurological studies, including the small number of participants and a lack of naturalistic environments, such that the ecological validity of the results has been questioned [36]. Studies of the musical brain should generally take place in highly controlled and somewhat culturally impoverished environments [37].

There is also an intense debate on how the brain perceives musical sounds. For instance, some authors claim a strong role for environmental effects: musical perception can change depending on the environment to which we are exposed. Research on brain plasticity shows that neuronal responses to frequency and other features depend on the sounds that an individual is exposed to early in life [38]. McDermott et al [39] have investigated the Tsimane, a native and isolated community living in the Amazon forest with minimal exposure to Western culture, who have not shown preference for the intervals considered consonants over dissonant ones (in our tempered system). This instance supports the proposition that perception is culturally conditioned by listening: people often listen to music that do not choose, to which they do not pay directed attention, but it is cognitively processed. The Tsimane find consonant and dissonant chords and vocal harmonies equally pleasant. In contrast, Bolivian city- and town-dwellers exhibited significant preferences for consonance, albeit to a lesser degree than United States residents. The results indicate that consonance preferences can be absent in cultures sufficiently isolated from Western music and are thus unlikely to reflect innate biases or exposure to harmonic natural sounds. In this sense, recent studies demonstrated the effects of musical training on the brain responses to music (e.g.[40,41] and how subjects' expectations modulate musical responses differently depending on their cultural experience and musical exposure [42].

Psychophysiology and music

Since ancient times, music and dance has been used as treatment of some diseases, including epilepsy and movement disorders (dyskinesia, chorea, etc.). Dances like Dionysia in Ancient Greece, St. Vitus dance in the Middle Ages and

tarantism, for example, were considered curative rituals for neurological and psychiatric conditions (see Sironi & Riva [43]. However, what is the biological role of music in brain disorders? Scientific research has contributed to shed light on many questions about the impact of music in human body and brain.

Music seems to elicit emotions and change moods through its stimulation of the autonomic nervous system (Hallam 2015). Whether the neural basis of the emotional appraisal of music is innate or acquired through acculturation remains a subject of discussion (Peretz & Sloboda 2005). Several neuroscientific approaches to music and emotion have focused on to the structure and function of neural correlates of music-evoked emotions. Kreutz et al. [44] presented 25 classical music excerpts representing 'happiness', 'sadness', 'fear', 'anger', and 'peace', to listeners who rated each excerpt for emotion, valence, and arousal. Ratings were entered into a parametric modulation analysis of activations in the entire brain. Results showed that valence as well as positive emotions were associated with activations in cortical and limbic areas, including the anterior cingulum, basal ganglia, insula, and nucleus accumbent. Negative emotions, however, did not yield significant activations at group level [44].

Koelsch [45] argued that most neuroscience studies on emotion have used static visual images, while details about how pleasant emotions unfold over time, remain to be explored. Music is particularly appropriate to investigate the time course of emotion throughout the length of musical stimuli. According to Kreutz & Lotze [46] there is a lack of empirical evidence relating environmental mediated influences on musical emotions. Baumgartner et al. [47] presented the first neurological study on emotions by examining the influence of visual and musical stimuli on brain processing. Musical impulse revealed the largest Alpha-Power activity (reflecting reduced brain activity) and a higher emotional intensity (according to psychological and physiological indicators).

Several studies have focused on how listening to music can trigger a wide range of physiological effects, including changes in heart rate, respiration, blood pressure, skin conductivity, skin temperature, muscle tension, and biochemical responses. Krumhansl [48] obtained several physiological measures (including cardiac, vascular, electrodermal, and respiratory functions) from listeners of musical excerpts chosen to represent one of three emotions (sadness, fear, and happiness). As a result, the physiological reaction to music increased over time,

remarking the importance of music extension in clinical essays. The physiological measures all showed a significant effect of music compared to the pre-music interval. A few analyses, including correlations between physiology and emotion judgments, found significant differences among the excerpts. Thus, the sad excerpts produced the largest changes in heart rate, blood pressure, skin conductance and temperature. The fear excerpts produced the largest changes in blood transit time and amplitude. The happy excerpts produced the largest changes in the measures of respiration. These emotion-specific physiological changes only partially replicated those found for nonmusical emotions.

Respiration rate allows to differentiate between happy and sad excerpts, but this may be attributable to entrainment of respiration to the rhythm or the tempo rather than to emotions [49]. In order to test this hypothesis, Khalfa et al [50] investigated whether fast and slow rhythm or tempo are sufficient to induce differential physiological effects. Psychophysiological responses (electrodermal responses, facial muscles activity, blood pressure, heart, and respiration rate) were then measured in young adults listening to fast-happy and slow-sad music, and to two control versions of these excerpts created by removing pitch variations (rhythmic version) and both pitch and temporal variations (beat-alone). The results indicated that happy and sad music are significantly differentiated (happy > sad) by diastolic blood pressure, electrodermal activity, and zygomatic activity, while the fast and slow rhythmic and tempo control versions did not elicit such differentiations. In contrast, respiration rate was faster with stimuli presented at fast tempo relative to slow stimuli in the beat-alone condition.

In research by Carpentier y Potter [51], fast- and slow-paced rock and classical music excerpts were compared to silence. According to this study, skin conductance response (SCR) frequency was greater during music processing than during silence: fast-paced music elicits greater activation than slow-paced music. Genre significantly interacted with tempo in SCR frequency, with faster tempo increasing activation for classical music and decreasing it for rock music.

It has been also reported that music plays a particular role in the reduction of stress and anxiety, which in turn is connected to pain reduction and the strengthening of the immune system. A very early documented case of the use of music in the context of surgery is reported by a physician, Kane Evan O'Neil

[52], at the beginning of 20th century, indicating the benefits of using music within the operating room.

In another pioneering study, Guzzetta [53] found that music has a calming effect and may even be effective to reduce stress when used with cardiac patients. This study indicated that lowering apical heart rates and raising peripheral temperatures were more successful in the relaxation and music therapy groups than in a control group. The incidence of cardiac complications was found to be lower in the intervention groups, and most intervention subjects believed that such therapy was helpful. Davis and Thaut [54] surprisingly found physiological data that showed that the music aroused and excited rather than soothed autonomic and muscular activity, even though subjects reported decreases in state anxiety and increases in relaxation. Many subsequent investigations demonstrated the benefit of music on cardiomyopathies (e.g.,[55,56].

Listening to music triggered significant reductions in cortisol levels in healthy adults, a psychophysiological measure of stress [57-59]. Fukui & Yamashita [57] found that listening to Japanese popular songs decreased cortisol levels and decreased testosterone in males, whereas it increased testosterone in females.

In a more recent study, de Albuquerque et al. [60] claimed that, after the experience of listening to music while undergoing catheterization, 100% of the patients interviewed claimed they had overcome stress and felt calmness, tranquility, peace, and happiness. Some patients described the music as a companion, as something that diverts their attention from fear, transporting them to an imaginary place, to another dimension. The study by Kreutz et al. [58] indicated that listening to segments from Mozart's Requiem led to an increase in negative mood, a decrease in cortisol, and no significant changes in positive mood and secretory immunoglobulin A.

Mockel et al. (1994) concluded that different types of music induced cardiovascular, hormonal, and mental changes. After rhythmic music (Strauss's waltz) atrial filling fraction and atrial natriuretic peptide increased significantly, with an improvement of the mental state. Indian meditative music (composed by Ravi Shankar) lowered plasma cortisol, noradrenaline, and t-PA concentrations; Prolactin concentrations decreased after modern music (composed by H.W. Henze).

It has been shown that making or listening to music may also have an impact on the immune system [58,61]. In a naturalistic pre-post design carried out by Beck et al. (2000), samples of saliva were collected from members of a professional choir during an early rehearsal, a late rehearsal, and a public performance of Beethoven's *Missa Solemnis*. As measures of immune system response, mean levels of secretory immunoglobulin A increased significantly, as a proportion of whole protein, 150% during rehearsals and 240% during the performance. Meanwhile, cortisol concentrations decreased an average of 30% during rehearsals and increased 37% during performance. According to last review on the topic [62], a range of effects of music on neurotransmitters, hormones, cytokines, lymphocytes, vital signs, and immunoglobulins have been demonstrated.

Music can also play a therapeutic role in supporting rehabilitation of motor movements. Thaut et al [63] provided a scientific basis for the development of neurologic music therapy based on the temporal structure of music: “the periodicity of auditory rhythmic patterns could improve movement patterns in patients with movement disorders (…) mechanisms of rhythmic entrainment may be an essential tool for rehabilitation in all domains of neurologic music therapy”. Parkinson disease (PD) and brain injury or cerebral palsy have been two major foci for motor rehabilitation studies including music therapy. A recent review by Devlin et al. [64] outlined the short-term benefits of rhythmic auditory stimulation on gait parameters including gait freezing in Parkinson disease.

Besides music listening, performing music can enhance motor rehabilitation too. The use of music-based movement has reported positive evidence in many clinical trials on treatment of motor function in PD [65]. Alves-Pinto et al. [66] found scientific evidence that supports that active musical instrument playing may be an efficient means for triggering rehabilitation with motor disorders resulting from brain damage. Although a moderate number of studies have been carried out to date, a systematic review [67] on music interventions on acquired brain injury advocated that more high-quality randomized controlled trials are needed because many of these studies present a high risk of bias.

Other neuroscientific research focused on cognitive rehabilitation, with special attention on the connection between music and memory. Samson and Peretz (2005) reported on an amnesic patient with bilateral lesions of the

temporal lobe and observed that repeated exposure to melodies enhanced both linking and recognition judgments. With regards to dementia, a condition affecting about 50 million people worldwide, recent studies [68,69] have noted the scarcity of rigorous scientific investigation on the impact of music in this medical condition, and current interpretations of the data are hampered by the lack of methodological rigor.

Epilepsy is a common neurological condition affecting around 65 million people worldwide, with a prevalence of 1 in 100 [70]. Interest in the link between music and epilepsy has increased in the last two decades. Research has involved attempts at converting brain waves in seizure and non- seizure states into music to examine its utility as a potential nondrug therapy in epilepsy. Music appears to have a divergent effect on patients with epilepsy [71].

Very recent research by Rafiee et al. [72] has indicated the positive effects of Mozart music on epilepsy patients, after a treatment period of three months of daily listening to the first six minutes of *Sonata for two pianos in D Major* (Mozart K.448). The results showed daily listening to Mozart was associated to a reduction of seizure frequency in adult individuals. Previous research on children by the Royal Hospital for Sick Children [73] showed that children who listened to the first five minutes of Mozart's *Sonata for Two Pianos in D Major* had a significant reduction in the frequency of epileptic discharges, compared to those listening to control music.

Music is commonly used in pediatric settings to enhance the wellbeing of young patients. Some research has focused on the impact of active engagement with music on children who are hospitalized. Klassen et al (2008) published a systematic review of the impact of music to release the pain and anxiety in children undergoing clinical procedures, concluding that music, have shown clinical importance in reducing pharmaceutical interventions.

According to Preti & Welch [74], there is evidence of the musical intervention helping the children and their families to focus their attention on something that is external to the illness, because music constitutes a psychosocial space where they can interact without the anxiety and stress elicited by diagnosis-feared perception as well as illness. However, a recent review [75] highlights that more rigorous research is still needed.

Anderson & Patel [76] provide a framework for considering how music might attenuate stress in neonatal intensive care unit infants, and its effects on the cardio-pulmonary system and behavior of neonates. Also Thiel et al. [77] , demonstrate how music has positive effects on neurological development, on basic vital signs and on the reduction of pain in neonates.

Amusia

Congenital amusia is a lifelong impairment of music perception and memory that affects 4% of the population [78]. Amusia is a central auditory processing disorder characterized by the inability to discriminate pitch, reproduce melodies or to recognize deviations in melodic structure, in spite of normal hearing [79]. Amusia can manifest in different ways: for example, it can affect the perception of the tone, that is, those suffering from amusia are unable to perceive the relative pitch of the notes; it can also affect to the ability to perceive rhythm. Tranchant and Peretz [80] suggest that the beat impairment of amusia subjects is due to an imprecise internal timekeeping mechanism.

Deficits in this musical pitch perception have been shown to be strongly genetic in origin. A pioneer pedigree and twin study [78] discovered a high heritability of (70–80%) of amusia. The study by Peretz et al. [81] is the first systematic search of familial aggregation of congenital amusia; the authors indicated that 39% of first-degree relatives of families with this condition shared the same disorder, compared to only 3% of control families. The results clearly point to a strongly inherited component of amusia, suggesting that congenital amusia has a strong genetic basis.

Due to the lack of studies carried out from genetics, we here highlight the findings of neurosciences to point out the importance of this topic. According to Lagrois and Peretz [82], congenital amusia represents a rare opportunity to study the neurobiology of music cognition by tracing causal links between genes, brain, and behavior. In this regard, behavioral and neuropsychological studies have suggested that tonal and verbal short-term memory are supported by specialized neural networks. However, only a few neuroimaging investigations support this hypothesis. Albouy et al. [83] investigated the hypothesis of the existence of distinct neural resources for tonal and verbal memory by comparing typical non-musician listeners to individuals with congenital amusia, who exhibit pitch

memory impairments with preserved verbal memory. Their results suggest the existence of specialized cortical systems for short-term tonal and verbal memory in the human brain. Their findings represent an apparent contradiction: While the results provide a potential electrophysiological substrate for auditory unawareness (that is the hallmark of amusia), they also suggest that tone deaf subjects are processing musical abnormalities that they cannot consciously perceive. Jasmin et al. [84] found that individuals with amusia exhibited decreased functional connectivity between left frontal areas and right hemisphere pitch-related regions.

Amusia can also be acquired, and there are many cases described in the literature on neurological disorders. A well-known case is that of the composer Maurice Ravel (1875-1937). Towards the end of his life, he suffered a neurological disease with affections in the prefrontal cortex and the basal ganglia, which produced a Wernicke aphasia. His musical abilities were preserved, even though his writing and reading abilities were strongly affected [85].

References

1. Altenmüller, E.; Finger, S.; Boller, F. *Music, Neurology, and Neuroscience: Evolution, the Musical Brain, Medical Conditions, and Therapies Progress in Brain Research*; Amsterdam: Elsevier: 2015; Vol. 217, pp. 143-154.
2. Harvey, A.R. Music and the Meeting of Human Minds. *Front Psychol* **2018**, *9*, 762, doi:10.3389/fpsyg.2018.00762.
3. Perrone-Capano, C.; Volpicelli, F.; di Porzio, U. Biological bases of human musicality. *Rev Neurosci* **2017**, *28*, 235-245, doi:10.1515/revneuro-2016-0046.
4. Brown, S. *The “musilanguage” model of music evolution*; Cambridge: Bradford Books: 2001.
5. Peretz, I. Music, language and modularity framed in action. *Psychol. Belg.* **2009**, *49*, 157-175.
6. Wallin, N.L.; Merker, B.; Brown, S. *The origins of music*; Cambridge: Bradford Books: 2001.
7. Whaling, C. *What´s behind a song? The Neural Basis of song learning in birds*; Cambridge: Bradford Books: 2001.

8. Slater, P. *Birdsong Repertoires: The origins and use*; Cambridge: Bradford Books: 2001.
9. Marler, P. *Origins of music and speech: Inside from animals*; Cambridge: Bradford Books: 2001.
10. Lattenkamp, E.Z.; Vernes, S.C.; Wiegrebe, L. Volitional control of social vocalisations and vocal usage learning in bats. *J Exp Biol* **2018**, *221*, doi:10.1242/jeb.180729.
11. Fishman, Y.I.; Volkov, I.O.; Noh, M.D.; Garell, P.C.; Bakken, H.; Arezzo, J.C.; Howard, M.A.; Steinschneider, M. Consonance and dissonance of musical chords: neural correlates in auditory cortex of monkeys and humans. *J Neurophysiol* **2001**, *86*, 2761-2788, doi:10.1152/jn.2001.86.6.2761.
12. Crespo-Bojorque, P.; Monte-Ordono, J.; Toro, J.M. Early neural responses underlie advantages for consonance over dissonance. *Neuropsychologia* **2018**, *117*, 188-198, doi:10.1016/j.neuropsychologia.2018.06.005.
13. Peretz, I. The nature of music from a biological perspective. *Cognition* **2006**, *100*, 1-32, doi:10.1016/j.cognition.2005.11.004.
14. Johansson, B.B. Brain plasticity in health and disease. *Keio J Med* **2004**, *53*, 231-246, doi:10.2302/kjm.53.231.
15. Peñalba, A. La defensa de la educación musical desde las neurociencias. *Revista Electrónica Complutense de Investigación en Educación Musical* **2017**, *14*, 109-127.
16. Peretz, I. Processing of local and global musical information by unilateral brain-damaged patients. *Brain : a journal of neurology* **1990**, *113 (Pt 4)*, 1185-1205, doi:10.1093/brain/113.4.1185.
17. Peretz, I.; Kolinsky, R. Boundaries of separability between melody and rhythm in music discrimination: a neuropsychological perspective. *Q J Exp Psychol A* **1993**, *46*, 301-325, doi:10.1080/14640749308401048.
18. Merker, B. *Synchronous chorusing and human origins*; Cambridge: Bradford Books: 2001.
19. Wieser, H.G. Music and the brain. Lessons from brain diseases and some reflections on the "emotional" brain. *Ann N Y Acad Sci* **2003**, *999*, 76-94, doi:10.1196/annals.1284.007.

20. Schlaug, G.; Jancke, L.; Huang, Y.; Staiger, J.F.; Steinmetz, H. Increased corpus callosum size in musicians. *Neuropsychologia* **1995**, *33*, 1047-1055, doi:10.1016/0028-3932(95)00045-5.
21. Keenan, J.P.; Thangaraj, V.; Halpern, A.R.; Schlaug, G. Absolute pitch and planum temporale. *Neuroimage* **2001**, *14*, 1402-1408, doi:10.1006/nimg.2001.0925.
22. Elmer, S.; Rogenmoser, L.; Kuhnis, J.; Jancke, L. Bridging the gap between perceptual and cognitive perspectives on absolute pitch. *J Neurosci* **2015**, *35*, 366-371, doi:10.1523/JNEUROSCI.3009-14.2015.
23. Kumar, S.; Sedley, W.; Barnes, G.R.; Teki, S.; Friston, K.J.; Griffiths, T.D. A brain basis for musical hallucinations. *Cortex* **2014**, *52*, 86-97, doi:10.1016/j.cortex.2013.12.002.
24. Zabalza-Estévez, R.J. Musical hallucinations: perpetual music. *Rev. Neurol.* **2014**, *58*, 207-212.
25. Evers, S.; Ellger, T. The clinical spectrum of musical hallucinations. *J Neurol Sci* **2004**, *227*, 55-65, doi:10.1016/j.jns.2004.08.004.
26. Blood, A.J.; Zatorre, R.J. Intensely pleasurable responses to music correlate with activity in brain regions implicated in reward and emotion. *Proc Natl Acad Sci U S A* **2001**, *98*, 11818-11823, doi:10.1073/pnas.191355898.
27. Thaut, M.H. *Music as therapy in early history*; 2015; Vol. 217.
28. Quintiliano, A. *Sobre la música*, Traducción de Luis Colomer y Begoña Gil ed.; Madrid: Gredos: 1996.
29. Quintilianus, A. *On music in three Books. translation with introduction, commentary, and annotations by Thomas J. Mathiesen*; New Haven: Yale University Press: 1983.
30. Anderson, W.; Thomas, J.M. Ethos. In *The new grove dictionary of music and musicians*, Sadie, S., Tyrrell, J., Eds. Macmillan Publisher: London, 2001.
31. Anderson, W.; Thomas, J.M. Damon. In *The new grove dictionary of music and musicians*, Sadie, S., Tyrrell, J., Eds. Macmillan Publisher: London, 2001.
32. Mattheson, J.; Lenneberg, H. Johann Mattheson on affect and rhetoric in music (I). *J Music Ther* **1958**, *2*, 47-84.

33. Zatorre, R.; McGill, J. Music, the food of neuroscience? *Nature* **2005**, *434*, 312-315, doi:10.1038/434312a.
34. Beaty, R.E. The neuroscience of musical improvisation. *Neurosci Biobehav Rev* **2015**, *51*, 108-117, doi:10.1016/j.neubiorev.2015.01.004.
35. Pinho, A.L.; de Manzano, O.; Fransson, P.; Eriksson, H.; Ullen, F. Connecting to create: expertise in musical improvisation is associated with increased functional connectivity between premotor and prefrontal areas. *J Neurosci* **2014**, *34*, 6156-6163, doi:10.1523/JNEUROSCI.4769-13.2014.
36. Beaty, R.E.; Benedek, M.; Silvia, P.J.; Schacter, D.L. Creative cognition and brain network dynamics. *Trends Cogn Sci* **2016**, *20*, 87-95, doi:10.1016/j.tics.2015.10.004.
37. Peretz, I.; Sloboda, J.A. Music and the emotional brain. *Ann. N. Y. Acad. Sci.* **2005**, *1060*, 409-411.
38. Schreiner, C.E.; Polley, D.B. Auditory map plasticity: diversity in causes and consequences. *Curr Opin Neurobiol* **2014**, *24*, 143-156, doi:10.1016/j.conb.2013.11.009.
39. McDermott, J.H.; Schultz, A.F.; Undurraga, E.A.; Godoy, R.A. Indifference to dissonance in native Amazonians reveals cultural variation in music perception. *Nature* **2016**, *535*, 547-550, doi:10.1038/nature18635.
40. Jentschke, S.; Koelsch, S. Musical training modulates the development of syntax processing in children. *Neuroimage* **2009**, *47*, 735-744, doi:10.1016/j.neuroimage.2009.04.090.
41. Oechslin, M.S.; Van De Ville, D.; Lazeyras, F.; Hauert, C.A.; James, C.E. Degree of musical expertise modulates higher order brain functioning. *Cereb Cortex* **2013**, *23*, 2213-2224, doi:10.1093/cercor/bhs206.
42. Di Liberto, G.M.; Pelofi, C.; Bianco, R.; Patel, P.; Mehta, A.D.; Herrero, J.L.; de Cheveigne, A.; Shamma, S.; Mesgarani, N. Cortical encoding of melodic expectations in human temporal cortex. *Elife* **2020**, *9*, doi:10.7554/eLife.51784.
43. Sironi, V.A.; Riva, M.A. Neurological implications and neuropsychological considerations on folk music and dance. *Prog Brain Res* **2015**, *217*, 187-205, doi:10.1016/bs.pbr.2014.11.027.

44. Kreutz, G.; Ott, U.; Wehrum, S. Cerebral correlates of musically-induced emotions: an fMRI-study. In Proceedings of Proceedings of the 9ª International Conference on Music Perception and Cognition (ICMPC), Bologna, 22-26.
45. Koelsch, S. Investigating emotion with music: neuroscientific approaches. *Ann N Y Acad Sci* **2005**, *1060*, 412-418, doi:10.1196/annals.1360.034.
46. Kreutz, G.; Lotze, M. *Neuroscience of music and emotions*; Nova Science Publishers: 2007.
47. Baumgartner, T.; Esslen, M.; Jäncke, L. From emotion perception to emotion experience: emotions evoked by pictures and classical music. *Int J Psychophysiol* **2006**, *60*, 34-43.
48. Krumhansl, C.L. An exploratory study of musical emotions and psychophysiology. *Can J Exp Psychol* **1997**, *51*, 336-353, doi:10.1037/1196-1961.51.4.336.
49. Etzel, J.A.; Johnsen, E.L.; Dickerson, J.; Tranel, D.; Adolphs, R. Cardiovascular and respiratory responses during musical mood induction. *Int J Psychophysiol* **2006**, *61*, 57-69, doi:10.1016/j.ijpsycho.2005.10.025.
50. Khalfa, S.; Roy, M.; Rainville, P.; Dalla Bella, S.; Peretz, I. Role of tempo entrainment in psychophysiological differentiation of happy and sad music? *Int J Psychophysiol* **2008**, *68*, 17-26, doi:10.1016/j.ijpsycho.2007.12.001.
51. Carpentier, F.; Potter, R. Effects of music on physiological arousal: Explorations into tempo and genre. *Media Psychol* **2007**, *10*, 339-363.
52. K.E., O.N. The psychology of anesthesia. *Current Researches in Anesthesia & Analgesia* **1930**, *9*, 236-238.
53. Guzzetta, C.E. Effects of relaxation and music therapy on patients in a coronary care unit with presumptive acute myocardial infarction. *Heart Lung* **1989**, *18*, 609-616.
54. Davis, W.B.; Thaut, M.H. The influence of preferred relaxing music on measures of state anxiety, relaxation, and physiological responses *J Music Ther* **1989**, *26*, 168-187.
55. Bradt, J.; Dileo, C.; Potvin, N. Music for stress and anxiety reduction in coronary heart disease patients. *The Cochrane database of systematic*

reviews **2013**, 10.1002/14651858.CD006577.pub3, CD006577, doi:10.1002/14651858.CD006577.pub3.

56. Shen, B.J.; Fan, Y.; Lim, K.S.C.; Tay, H.Y. Depression, anxiety, perceived stress, and their changes predict greater decline in physical health functioning over 12 months among patients with coronary heart disease. *Int J Behav Med* **2019**, *26*, 352-364, doi:10.1007/s12529-019-09794-3.
57. Fukui, H.; Yamashita, M. The effects of music and visual stress on testosterone and cortisol in men and women. *Neuro Endocrinol Lett* **2003**, *24*, 173-180.
58. Kreutz, G.; Bongard, S.; Rohrmann, S.; Hodapp, V.; Grebe, D. Effects of choir singing or listening on secretory immunoglobulin A, cortisol, and emotional state. *J Behav Med* **2004**, *27*, 623-635, doi:10.1007/s10865-004-0006-9.
59. Mockel, M.; Rocker, L.; Stork, T.; Vollert, J.; Danne, O.; Eichstadt, H.; Muller, R.; Hochrein, H. Immediate physiological responses of healthy volunteers to different types of music: cardiovascular, hormonal and mental changes. *Eur J Appl Physiol Occup Physiol* **1994**, *68*, 451-459, doi:10.1007/BF00599512.
60. de Albuquerque Paiva, S.; de Oliveira Moreira, J.; Silveira, F.R. Feelings and senses given to the music present at the hospital during hemodynamic procedures: Cardiac catheterizationand coronary angioplasty. *Open J Med Psychol* **2017**, *6*, 31-51.
61. Beck, R.; Cesario, T.; Yousefi, S.; Enamoto, H. Choral singing, performance perception and immune system changes in salivary immunoglobulin and cortisol. *Music Percept.* **2000**, *18*, 87-106.
62. Fancourt, D.; Ockelford, A.; Belai, A. The psychoneuroimmunological effects of music: a systematic review and a new model. *Brain Behav Immun* **2014**, *36*, 15-26, doi:10.1016/j.bbi.2013.10.014.
63. Thaut, M.H.; McIntosh, G.C.; Hoemberg, V. Neurobiological foundations of neurologic music therapy: rhythmic entrainment and the motor system. *Front Psychol* **2014**, *5*, 1185, doi:10.3389/fpsyg.2014.01185.

64. Devlin, K.; Alshaikh, J.T.; Pantelyat, A. Music therapy and music-based interventions for movement disorders. *Curr Neurol Neurosci Rep* **2019**, *19*, 83, doi:10.1007/s11910-019-1005-0.
65. Zhang, S.; Liu, D.; Ye, D.; Li, H.; Chen, F. Can music-based movement therapy improve motor dysfunction in patients with Parkinson's disease? Systematic review and meta-analysis. *Neurol Sci* **2017**, *38*, 1629-1636, doi:10.1007/s10072-017-3020-8.
66. Alves-Pinto, A.; Turova, V.; Blumenstein, T.; Lampe, R. The case for musical instrument training in cerebral palsy for neurorehabilitation. *Neural Plast* **2016**, *2016*, 1072301, doi:10.1155/2016/1072301.
67. Magee, W.L.; Clark, I.; Tamplin, J.; Bradt, J. Music interventions for acquired brain injury. *The Cochrane database of systematic reviews* **2017**, *1*, CD006787, doi:10.1002/14651858.CD006787.pub3.
68. Baird, A.; Samson, S. *Music and dementia*; Amsterdam: Elsevier: 2015; Vol. 217.
69. Nair, B.R.; Browne, W.; Marley, J.; Heim, C. Music and dementia. *Degener Neurol Neuromuscul Dis* **2013**, *3*, 47-51, doi:10.2147/DNND.S35762.
70. Thurman, D.J.; Beghi, E.; Begley, C.E.; Berg, A.T.; Buchhalter, J.R.; Ding, D.; Hesdorffer, D.C.; Hauser, W.A.; Kazis, L.; Kobau, R., et al. Standards for epidemiologic studies and surveillance of epilepsy. *Epilepsia* **2011**, *52 Suppl 7*, 2-26, doi:10.1111/j.1528-1167.2011.03121.x.
71. Maguire, M. *Music and its association with epileptic disorders*; Amsterdam: Elsevier, 2015; Vol. 217.
72. Rafiee, M.; Patel, K.; Groppe, D.M.; Andrade, D.M.; Bercovici, E.; Bui, E.; Carlen, P.L.; Reid, A.; Tai, P.; Weaver, D., et al. Daily listening to Mozart reduces seizures in individuals with epilepsy: A randomized control study. *Epilepsia Open* **2020**, *5*, 285-294, doi:10.1002/epi4.12400.
73. Grylls, E.; Kinsky, M.; Baggott, A.; Wabnitz, C.; McLellan, A. Study of the Mozart effect in children with epileptic electroencephalograms. *Seizure* **2018**, *59*, 77-81, doi:10.1016/j.seizure.2018.05.006.
74. Preti, C.; Welch, G.F. Music in a hospital: The impact of a live music program on pediatric patients and their caregivers. *Music and Medicine* **2011**, *3*, 213-123.

75. Stegemann, T.; Geretsegger, M.; Phan Quoc, E.; Riedl, H.; Smetana, M. Music Therapy and Other Music-Based Interventions in Pediatric Health Care: An Overview. *Medicines (Basel)* **2019**, *6*, doi:10.3390/medicines6010025.
76. Anderson, D.E.; Patel, A.D. Infants born preterm, stress, and neurodevelopment in the neonatal intensive care unit: might music have an impact? *Dev Med Child Neurol* **2018**, *60*, 256-266, doi:10.1111/dmcn.13663.
77. Thiel, M.T.; Findeisen, B.; Langler, A. Music therapy as part of integrative neonatology: 20 years of experience - 3 case reports and a review. *Forsch Komplementmed* **2011**, *18*, 31-35, doi:10.1159/000323714.
78. Kalmus, H.; Fry, D.B. On tune deafness (dysmelodia): frequency, development, genetics and musical background. *Ann. Hum. Genet.* **1980**, *43*, 369-382.
79. Braun, A.; McArdle, J.; Jones, J.; Nechaev, V.; Zalewski, C.; Brewer, C.; Drayna, D. Tune deafness: processing melodic errors outside of conscious awareness as reflected by components of the auditory ERP. *PLoS One* **2008**, *3*, e2349, doi:10.1371/journal.pone.0002349.
80. Tranchant, P.; Peretz, I. Basic timekeeping deficit in the Beat-based Form of Congenital Amusia. *Scientific reports* **2020**, *10*, 8325, doi:10.1038/s41598-020-65034-9.
81. Peretz, I.; Cummings, S.; Dube, M.P. The genetics of congenital amusia (tone deafness): a family-aggregation study. *Am. J. Hum. Genet.* **2007**, *81*, 582-588, doi:10.1086/521337.
82. Lagrois, M.E.; Peretz, I. The co-occurrence of pitch and rhythm disorders in congenital amusia. *Cortex* **2019**, *113*, 229-238, doi:10.1016/j.cortex.2018.11.036.
83. Albouy, P.; Peretz, I.; Bermudez, P.; Zatorre, R.J.; Tillmann, B.; Caclin, A. Specialized neural dynamics for verbal and tonal memory: fMRI evidence in congenital amusia. *Hum Brain Mapp* **2019**, *40*, 855-867, doi:10.1002/hbm.24416.

84. Jasmin, K.; Dick, F.; Stewart, L.; Tierney, A.T. Altered functional connectivity during speech perception in congenital amusia. *Elife* **2020**, *9*, doi:10.7554/eLife.53539.

85. Warren J. 2003. Maurice Ravel's amusia. *J R Soc Med* 96:424. 10.1258/jrsm.96.8.424-a
